# Cell cycle exit during bortezomib‐induced osteogenic differentiation of mesenchymal stem cells was mediated by Xbp1s‐upregulated p21^Cip1^ and p27^Kip1^

**DOI:** 10.1111/jcmm.15605

**Published:** 2020-07-06

**Authors:** Dan Zhang, Rong Fan, Li Lei, Lei Lei, Yanmeng Wang, Nan Lv, Ping Chen, Ramone A. Williamson, Baiyan Wang, Jinsong Hu

**Affiliations:** ^1^ Department of Cell Biology and Genetics Xi’an Jiaotong University Health Science Center Xi’an China; ^2^ Department of Clinical Hematology Second Affiliated Hospital Xi'an Jiaotong University Health Science Center Xi'an China; ^3^ Key Laboratory of Environment and Genes Related to Diseases (Xi’an Jiaotong University) Ministry of Education Xi'an China

**Keywords:** bortezomib, cell cycle, mesenchymal stem cells, p21^Cip1^, p27^Kip1^, Xbp1s

## Abstract

Mesenchymal stem cells (MSCs) are multipotent cells capable of differentiating into a variety of cell types. Bortezomib, the first approved proteasome inhibitor used for the treatment of multiple myeloma (MM), has been shown to induce osteoblast differentiation, making it beneficial for myeloma bone disease. In the present study, we aimed to investigate the effects and underlying mechanisms of bortezomib on the cell cycle during osteogenic differentiation. We confirmed that low doses of bortezomib can induce MSCs towards osteogenic differentiation, but high doses are toxic. In the course of bortezomib‐induced osteogenic differentiation, we observed cell cycle exit characterized by G_0_/G_1_ phase cell cycle arrest with a significant reduction in cell proliferation. Additionally, we found that the cell cycle exit was tightly related to the induction of the cyclin‐dependent kinase inhibitors p21^Cip1^ and p27^Kip1^. Notably, we further demonstrated that the up‐regulation of p21^Cip1^ and p27^Kip1^ is transcriptionally dependent on the bortezomib‐activated ER stress signalling branch Ire1α/Xbp1s. Taken together, these findings reveal an intracellular pathway that integrates proteasome inhibition, osteogenic differentiation and the cell cycle through activation of the ER stress signalling branch Ire1α/Xbp1s.

## 1 INTRODUCTION

Stem cells possess a unique capacity for self‐renewal and can differentiate into multi‐lineage cells. Mesenchymal stem cells (MSCs) are adult stem cells originally isolated from mouse bone marrow, which exhibit plastic adherence properties and form spindle‐shaped colonies upon culture.^1^, ^2^ MSCs exhibit the ability to differentiate into specialized cells developing from mesoderm and even to transdifferentiate into ectodermal and endodermal lineages. Moreover, in recent years, MSCs have been shown to possess immunomodulatory properties; these observations have sparked interest in the potential application of MSCs for cell‐based therapy in regenerative medicine and immune diseases.^3^, ^4^ Nonetheless, our understanding of the mechanisms by which MSCs impact clinical and immunological abnormalities in these diseases remains incomplete. For instance, MSCs have been suggested to be attracted to primary tumours, thereby contributing to tumour metastasis as well as drug resistance.^5^, ^6^, ^7^, ^8^, ^9^, ^10^ On the other hand, chemotherapeutic drug treatments have been shown to alter the phenotype and differentiation potential of MSCs, and even render them more chemoprotective of the tumour cells.^11^, ^12^, ^13^, ^14^ Accordingly, further therapeutic efforts to target MSCs may help to prevent chemoresistance and disease relapse in tumours.

The proteasome is a central component of the protein degradation machinery in eukaryotic cells. Inhibition of the proteasome has emerged as a powerful approach for the treatment of multiple myeloma (MM), a haematologic cancer characterized by the accumulation of malignant plasma cells in the bone marrow (BM).^15^ Bortezomib, as the first approved proteasome inhibitor, has been used as a first‐line drug for the treatment of MM.^16^ In addition to its direct antitumour activity, bortezomib also exerts bone protection effects in MM patients. Of note, the effect of bortezomib on bone formation has been suggested to be related to the enhanced differentiation of MSCs towards osteoblasts.^13^, ^17^, ^18^

Although the fate determination and terminal differentiation of MSCs are known to be tightly controlled by diverse transcription factors and signalling pathways, many observations have identified important connections between cell fate decisions and the cell cycle machinery in pluripotent stem cells.^19^, ^20^, ^21^ For example, terminal differentiation is usually associated with cell cycle exit, and the transition through mitosis and G_1_ phase plays an essential role in establishing a window of opportunity for pluripotency exit and the initiation of differentiation.^19^

The purpose of this study was to determine the mechanisms by which the bortezomib‐induced differentiation of MSCs towards osteoblasts affects the cell cycle machinery.

## 2 MATERIALS AND METHODS

### 2.1 Reagents and antibodies

Bortezomib was purchased from LC Laboratories (Woburn, MA, USA). For in vitro studies, the drug was reconstituted in dimethyl sulfoxide (DMSO) at a stock concentration of 1 mM and stored at −80°C. The small‐molecule inhibitors MKC3946 and GSK2606414 were bought from MedChemExpress (Monmouth Junction, NJ, USA). The stock solution for MKC3946 and GSK2606414 was prepared in DMSO at a concentration of 10 mM and stored at −20°C. The antibodies against p21^Cip1^, p27^Kip1^, X‐box‐binding protein 1 (Xbp1s), activating transcription factor 6 (Atf6), 78 kDa glucose‐regulated protein (Grp78), C/EBP homologous protein (Chop), cyclin D3, cyclin E1, cyclin‐dependent kinase 2 (CDK2), cyclin‐dependent kinase 4 (CDK4) and β‐actin were obtained from Proteintech (Wuhan, Hubei, China), and the antibody against activating transcription factor 4 (Atf4) was obtained from Santa Cruz Biotechnology (Dallas, TX, USA). All other chemicals were obtained from Sigma‐Aldrich (Burlington, MA, USA) unless otherwise specified.

### 2.2 mBM‐MSC isolation and expansion

Inbred male C57BL/6 mice aged 4‐6 weeks were purchased from the animal centre of Xi'an Jiaotong University, housed and treated according to conditions approved by the Ethical Committee for Animal Experiments of the Xi'an Jiaotong University Health Science Center (No. 2015‐123). In brief, individual mice were killed by cervical dislocation, and the whole body was soaked thoroughly with 70% ethanol solution for 2 min. Following dissection of the hind legs and vertebrae, all tissues were removed from around the bones and the bones placed in a Petri dish with 5 mL of Dulbecco's modified Eagle's medium (DMEM; HyClone, Logan, Utah, USA). The ligaments between femur and hip were cut, and the bone was cut below the ankle joint. The tibia was separated from the femur by bending slightly at the knee joint. Holding the femur/tibia with a sterile forceps, both epiphyses were then removed with a sterile scissors. The contents of the bones were then flushed with a 1‐mL syringe with a needle, into a Petri dish with 5 mL of medium. The medium was then aspirated and flushed several times to disperse the bone marrow cells. The vertebrae were then crushed with the backside of a 5‐mL syringe in 5 mL of medium. The cell suspensions were then filtered through a nylon filter (70 µM mesh diameter) into a 50‐mL tube. Pipet 5 mL of Lympholyte M (Cedarlane, Ontario, Canada) into a 15‐mL tube, and overlayed carefully with 5 mL of the cell suspension. The mixture was then centrifuged for 20 min at 1,000 g, and the cells at the interface of the Lympholyte M and medium were removed. The mononuclear cells were then washed two times in 5 mL of medium. Cells were counted, and the cell concentration was adjusted to 5 × 10^6^/mL in complete culture medium consisting of high‐glucose DMEM supplemented with 15% foetal bovine serum (FBS) (Biological Industries, Kibbutz Beit Haemek, Israel) and 100 U/mL penicillin‐streptomycin and 100 µg/mL L‐glutamine (Biosharp, Hefei, Anhui, China), and then plated in plastic 10‐cm plates. Non‐adherent cells were removed after 6 h, and the adherent cells were re‐fed with complete DMEM medium, with additional media changes every 3‐4 days. After approximately 8 days or when cell cultures reached confluence, cells were detached with 0.25% trypsin (w/v)/1 mM ethylenediaminetetraacetic acid (EDTA) solution (Biosharp, Hefei, Anhui, China) for 2 min and replated at 1 × 10^5^/mL, and regarded as passage 1 (P1) mBM‐MSCs. Passaging was performed every 4‐6 days at a split ratio of 1:3. P3‐P5 BM‐MSCs were used for all experiments.

### 2.3 Alizarin Red S staining

mBM‐MSCs were plated in 35‐mm‐diameter culture dishes and grown in DMEM containing 10% FBS, 100 U/mL penicillin, 100 μg/mL streptomycin, and 2 mM L‐glutamine at 37°C in a humidified incubator with 5% CO_2_ in the air. When cell density reached 70%‐80% confluence, the cells were subsequently cultured in the presence or absence of bortezomib at the indicated concentrations for 8 days. The culture medium was changed every 3 days. mBM‐MSCs cultured in an osteogenic induction media containing 50 mg/mL ascorbic acid and 10 mM β‐glycerophosphate sodium were used as a positive control. For Alizarin Red S (ARS) (Solarbio Life Sciences, Beijing, China) staining, mBM‐MSCs were fixed with 4% formaldehyde for 10 min, then rinsed twice with phosphatebuffered saline (PBS) at room temperature. The cells were then stained with 1% ARS (pH 4.2) for up to 15 min at room temperature. Images of mBM‐MSCs stained with ARS were captured and stored digitally.

### 2.4 MTT cell viability assay

mBM‐MSCs were seeded at a cell density of 5 × 10^3^ cells/mL in 96‐well plates and allowed to attach to the wells for 24 h. Cells were then treated with various concentrations of bortezomib. After 24‐h and 48‐h treatment, 10 µL of 3‐(4,5‐dimethylthiazol‐2‐yl)‐2,5‐diphenyltetrazolium bromide (MTT) solution (Sigma‐Aldrich, Burlington, MA, USA) was added to each well to a final concentration of 0.5 mg/mL. The supernatant medium was then discarded, and a volume of 150 µL of DMSO was added to each well to lyse the crystal. The plates were agitated for 15 min to ensure the dissolution of any remaining crystals. The absorbance in the relevant wells was measured at 490 nm using an Ultra Microplate Reader (ELx800, BioTek Instruments, Winooski, VT, USA). Each experiment was reproduced three times in duplicate.

### 2.5 EdU cell proliferation assay

5‐ethynyl‐2′‐deoxyuridine (EdU) incorporation assay was performed to measure cell proliferation with the kit (Molecular Probes, Eugene, OR, USA) according to the manufacturer's instructions. Briefly, mBM‐MSCs were seeded and grown at a density of 70%‐80% confluence in 35‐mm culture dishes and then treated with various concentrations of bortezomib for 24 h. EdU was added to the culture media 4 h before the harvest. The cells were detected by flow cytometry using a BD FACSCanto II instrument. Data were analysed using FlowJo 7.6.2 software (Tree Star, Ashland, OR, USA).

### 2.6 Cell cycle analysis

mBM‐MSCs (5 × 10^5^ cells) treated with various concentrations of bortezomib for 24 h were harvested, washed with PBS solution, fixed in 70% ethanol and kept at −20°C overnight. Cells were then resuspended in a buffer containing 50 mg/mL propidium iodide and 100 mg/mL RNase A (Solarbio Life Sciences, Beijing, China). Samples were analysed using a BD FACSCanto II flow cytometer. Data were acquired and analysed using CellQuest and ModFit software (BD Biosciences, San Jose, CA, USA).

### 2.7 RNA purification and Real‐time PCR analysis

Total RNA from cells was extracted using Ultrapure RNA Kit (DNase I) (CWBIO, Beijing, China) according to the manufacturer's instructions. Reverse transcription was performed using HiFiScript cDNA Synthesis Kit (CWBIO, Beijing, China). Real‐time PCR was performed using UltraSYBR Mixture (CWBIO, Beijing, China) and a CFX96™ Real‐Time PCR Detection System (Bio‐Rad, Hercules, CA, USA). The thermal cycling conditions included 2 min at 50°C and 10 min at 95°C, followed by 40 cycles of 95°C for 15 s, 58°C for 30 s and 72°C for 30 s. Mouse β‐actin was used as internal control. The relative expression levels of each gene were analysed using the 2^‐ΔΔCt^ method. The primers were designed using Primer3Plus platform (http://www.bioinformatics.nl/primer3plus) and synthesized by Sangon Biotech (Shanghai, China). The sequences of forward and reverse primers for these genes are listed in Table S1.

### 2.8 Western blotting analysis

Western blotting was performed as described previously.^16^ Briefly, mBM‐MSCs were cultured with indicated doses of bortezomib for 24 h; then, the cells were lysed in radioimmunoprecipitation assay (RIPA) buffer. Equal amount of proteins was loaded on 10% sodium dodecyl sulphate‐polyacrylamide gel electrophoresis (SDS‐PAGE) gel and was then electrotransferred to polyvinylidene difluoride (PVDF) membrane immunoblotted for specific primary antibodies. Immunoblots were visualized using horseradish peroxidase (HRP)‐conjugated secondary antibodies (Jackson ImmunoResearch Laboratories, West Grove, PA, USA) and the enhanced chemiluminescence (ECL) Western Blot Detection Kit (Phygene Life Sciences, Fuzhou, Fujian, China). The chemiluminescent blots were captured by using digital imaging with a charge‐coupled device (CCD) camera system MiniChemi 610 and analysed by Lane 1D™ analysis software (Sage, Beijing, China).

### 2.9 Lentiviral particle transduction

Full‐length human *XBP1s* cDNA was amplified and cloned into the Tet‐On inducible lentiviral vector GV437 (TetIIP‐MCS‐EGFP‐3FLAG‐Ubi‐TetR‐IRES‐Puromycin) (Genechem, Shanghai, China), and the construct sequence was verified by sequencing. Lentiviral particles were produced by standard transient transfection of a three‐plasmid system into producer cells 293T. One day before lentiviral infection, 1 × 10^5^ mBM‐MSCs in 2 mL cell growth medium were seeded in 35‐mm dishes, or 1 × 10^4^ mBM‐MSCs in 0.2 mL cell growth medium were seeded in 96‐well plate, adjusting the number of cells plated to accommodate a confluency of 50%‐60% upon transduction. After removal of culture medium, the lentivirus/polybrene mixture was added (for 35‐mm dish: 50 µL polybrene at 1 mg/mL, 100 µL 1 × 10^8^ TU/mL lentiviral particles, 850 µL cell culture medium; for 96‐well plate: 5 µL polybrene at 1 mg/mL, 10 µL 1 × 10^8^ TU/mL lentiviral particles, 85 µL cell culture medium) to the plated cells at multiplicity of infection (MOI) 100. The next day, the transduction efficiency was evaluated by monitoring green fluorescent protein (GFP) expression in living cells, and the lentivirus/polybrene mixture was replaced by fresh culture medium in the presence or absence of 2.5 μg/mL doxycycline to induce XBP1s expression in the transduced mBM‐MSCs. 24 h after the addition of doxycycline, the transduced cells were then harvested for further analysis.

### 2.10 Chromatin Immunoprecipitation

Chromatin immunoprecipitation (ChIP) was performed as described previously.^16^ Briefly, mBM‐MSCs treated with vehicle or 2.5 nM bortezomib for 16 h were cross‐linked with 1% formaldehyde. Fixation was then stopped by the addition of glycine to a final concentration of 0.125 M. After washing with ice‐cold PBS, the cells were lysed and nuclear extracts were prepared. Pelleted nuclei were then digested with micrococcal nuclease to produce 150‐ to 900‐bp DNA fragments. Then, the digested genomic DNA was immunoprecipitated with against control rabbit IgG or anti‐Xbp1s antibodies (BioLegend, San Diego, CA, USA) at 4°C overnight on a rocking platform, followed by incubation with the protein G magnetic beads (Cell Signaling Technology, Danvers, MA, USA). After washing, the immune complexes were eluted and were subjected to real‐time PCR analysis using primer pairs (Table S2) covering the putative regions of the *p21^Cip1^ and p27^Kip1^* promoters.

### 2.11 Statistical analysis

Results were statistically analysed in GraphPad Prism 5.0 (GraphPad Software Inc, San Diego, CA, USA) and presented as mean ± SEM. Statistically significant differences between two groups were assessed by two‐tailed unpaired t test. *P* < .05 was considered statistically significant.

## 3 RESULTS

### 3.1 Bortezomib decreases mBM‐MSC cell viability

**FIGURE 1:**
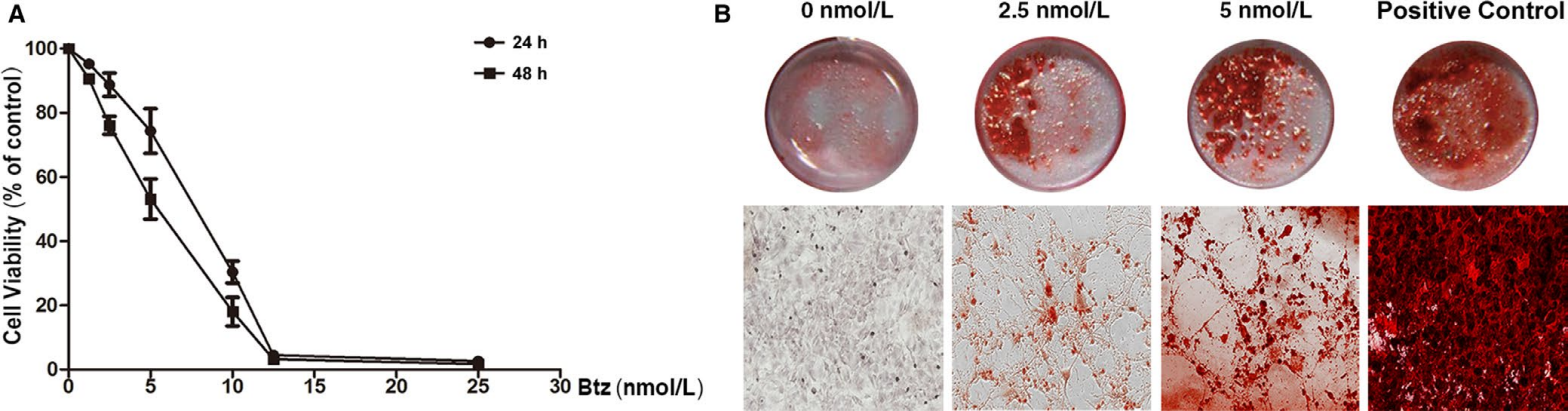
The effects of bortezomib (Btz) on the osteogenic differentiation of mBM‐MSCs. A, Bortezomib dose‐dependently inhibits mBM‐MSC cell viability. mBM‐MSCs were treated with serial doses of bortezomib for 1 or 2 days; the cell viability was then measured by MTT assay. B, Low doses of bortezomib induce osteogenic differentiation of mBM‐MSCs. mBM‐MSCs were cultured with bortezomib (0 nM, 2.5 nM, 5 nM) or in an osteogenic medium as a positive control for 8 days; then, the mineralized matrix formation was detected by ARS staining. Upper panel: representative images of whole plate; lower panel: representative images under microscope (10 × objective lens)

To assess the effects of bortezomib on cell viability, we performed MTT assay in mBM‐MSCs grown in various concentrations of bortezomib for 24 h and 48 h. As shown in Figure 1A, bortezomib dose‐dependently decreased the viability of mBM‐MSCs. The IC_50_ of bortezomib at 24 h and 48 h after treatment was 8.890 nM and 6.64 nM, individually. The data indicated that doses higher than 5 nM could result in cytotoxicity to mBM‐MSCs.

### 3.2 Low doses of bortezomib can induce osteogenesis in mBM‐MSCs

Bortezomib has been suggested to enhance bone formation in the bone marrow of MM patients. To see whether it could directly induce osteogenic differentiation of MSCs in vitro, we evaluated the effects of bortezomib on mBM‐MSCs. As the osteogenic differentiation of mBM‐MSCs ultimately leads to the deposition of calcium in the mineralized extracellular matrix, we evaluated calcium deposition in bortezomib‐treated mBM‐MSCs by ARS staining. As shown in Figure 1B, mBM‐MSCs were cultured for 8 days in the presence or absence of bortezomib, and ARS staining results showed that both 2.5 nM and 5 nM of bortezomib‐treated mBM‐MSCs deposited much higher amounts of calcium phosphate crystals than the control cells. To further prove the regulatory role of bortezomib in osteogenesis, we measured the changes of the other bone formation markers and confirmed that bortezomib can induce the expression of Runx2, Sp7, Col1A1, alkaline phosphatase (ALP) and osteocalcin (OCN/BGLAP) (Figure S1).

### 3.3 Bortezomib inhibits mBM‐MSC cell proliferation

**FIGURE 2:**
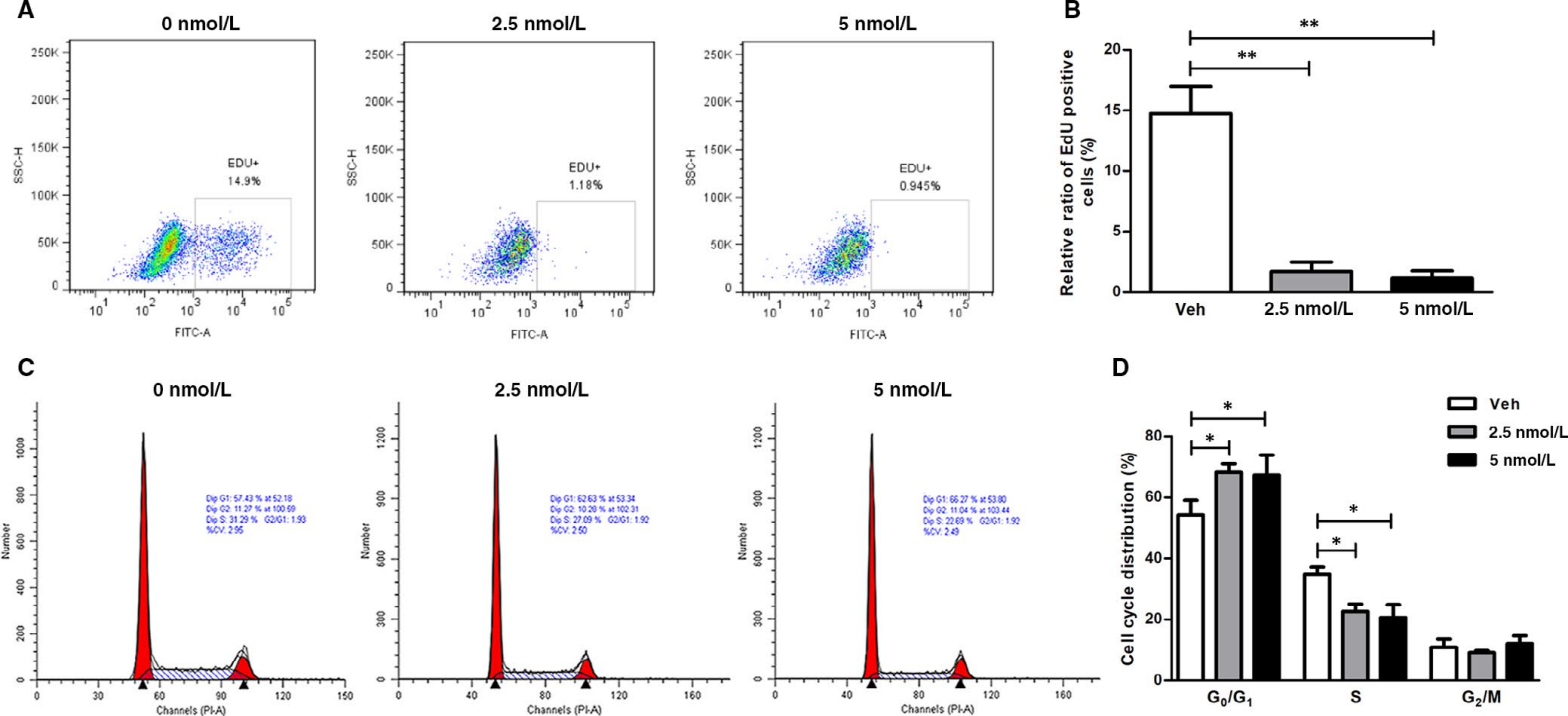
Bortezomib inhibits cell proliferation and induces G_0_/G_1_ cell cycle arrest in mBM‐MSCs. A, Flow cytometric analysis of the effects of bortezomib on cell proliferation of mBM‐MSCs. mBM‐MSCs were treated with indicated concentrations of bortezomib for 24 h; EdU incorporation assay was performed to evaluate cell proliferation by detecting the de novo‐synthesized DNA during S phase. B, Statistical analysis of the results of EdU incorporation assay. C, Bortezomib induces G_0_/G_1_ cell cycle arrest in mBM‐MSCs. mBM‐MSCs were treated with indicated doses of bortezomib for 24 h; then, the cells were collected, and the cell cycle was examined by flow cytometry. D, Statistical analysis of cell cycle distribution in response to bortezomib treatment. Data are presented as mean ± SEM of three independent experiments. * *P* < 0.05, ** *P* < 0.01

Given the potential association between cell differentiation and proliferation, we further investigated the effects of bortezomib on cell proliferation during bortezomib‐induced osteogenic differentiation. By using EdU incorporation assay, we found that bortezomib dose‐dependently decreased the numbers of EdU‐positive mBM‐MSCs, which represent the proliferating population (Figure 2A and B).

### 3.4 Bortezomib induces G_0_/G_1_ phase cell cycle arrest

Based on the finding above that bortezomib inhibits the proliferation of mBM‐MSCs, we further analysed the effect on the cell cycle distribution. As shown in Figure 2C and 2D, bortezomib treatment for 24 h significantly induced G_0_/G_1_ phase arrest in mBM‐MSCs. Compared with the control group, the percentages of G_0_/G_1_ phase of cells treated with 2.5 nM and 5 nM of bortezomib were increased from 55.14 ± 5.132 to 67.36 ± 6.067 and 68.117 ± 2.743, respectively. In contrast, the proportion of S phase cells were decreased from 32.017 ± 1.991 to 21.807 ± 2.844 and 19.940 ± 4.321. However, there was no significant change in the proportion of cells in the G_2_/M phase.

### 3.5 Bortezomib triggers changes in cell cycle machinery

**FIGURE 3:**
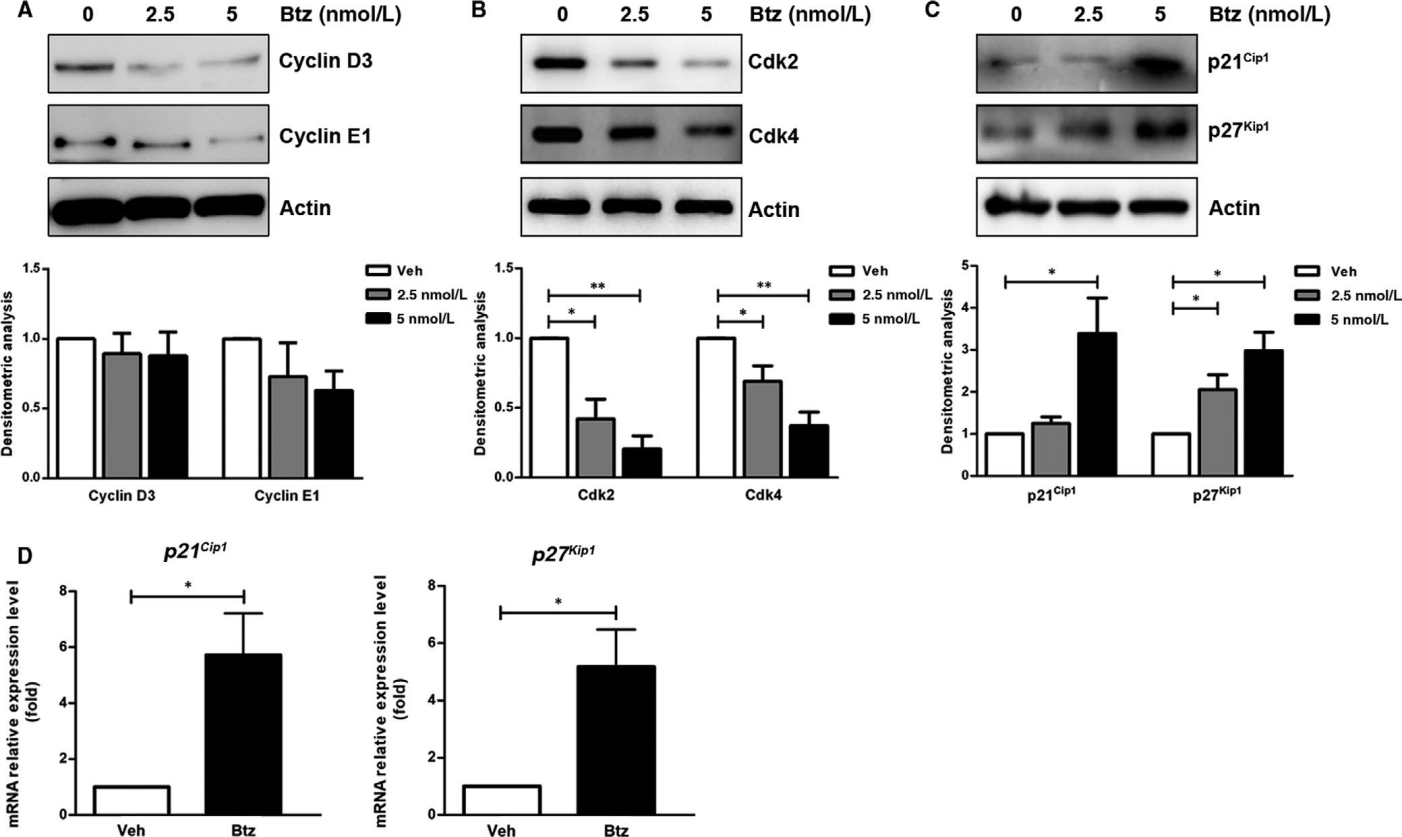
The effects of bortezomib on the expression of cell cycle regulatory components. (A‐C) Western blotting analysis of the expression of cyclins (A), CDKs (B) and CKIs (C) in mBM‐MSCs treated with indicated concentrations of bortezomib for 24 h. Upper panel: The protein levels of cyclins (cyclin D3 and E1), CDKs (Cdk2 and Cdk4) and CKIs (p21^Cip1^ and p27^Kip1^) were examined by Western blotting; lower panel: densitometric analysis of the Western blotting results from three independent experiments. (D) Real‐time PCR analysis of the expression of *p21^Cip1^* and *p27^Kip1^* on mRNA level in mBM‐MSCs treated with 2.5 nM of bortezomib for 24 h. The values represent the mean ± SEM of three experiments. * *P* < 0.05, ** *P* < 0.01, versus the control group

To further determine the molecular mechanism underlying G_0_/G_1_ phase cell cycle arrest, we examined the effects of bortezomib on the expression of G_0_/G_1_ phase‐associated cyclins, cyclin‐dependent kinases (CDKs) and cyclin‐dependent kinase inhibitors (CKIs). As shown in Figure 3A, bortezomib treatment has no effects on the expression of cyclin D3 and cyclin E1. However, the expression of Cdk2 and Cdk4 was markedly decreased by bortezomib (Figure 3B). In contrast, the expression of p21^Cip1^ and p27^Kip1^ was significantly increased by bortezomib (Figure 3C). In line with the increase in p21^Cip1^ and p27^Kip1^ at the protein level, we further observed that the mRNA levels of *p21^Cip1^* and *p27^Kip1^* were significantly up‐regulated by bortezomib (Figure 3D).

### 3.6 ER stress signalling Xbp1s is involved in the transcriptional regulation of bortezomib‐induced p21^Cip1^ and p27^Kip1^

**FIGURE 4:**
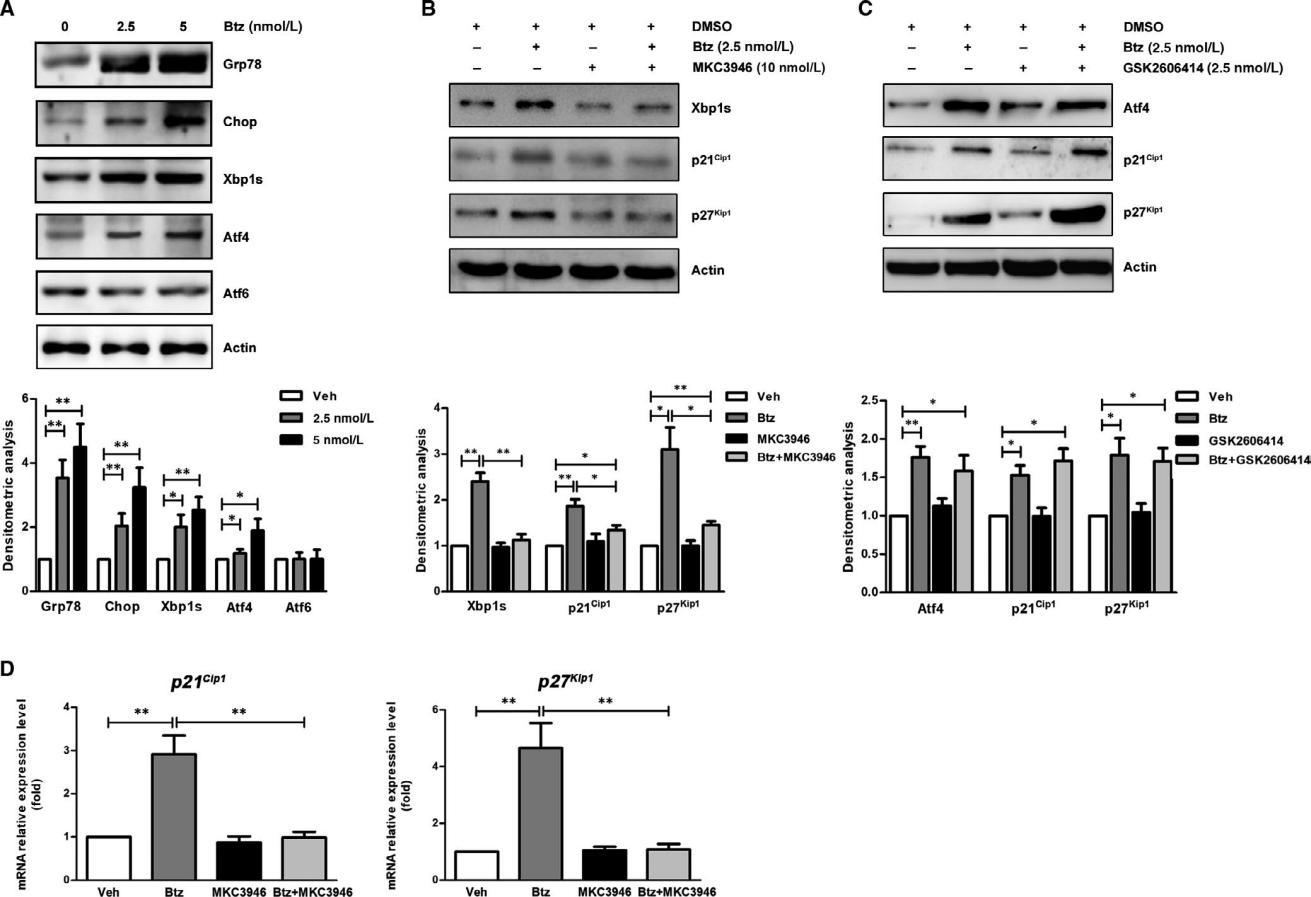
Bortezomib induces p21^Cip1^ and p27^Kip1^ expression through Xbp1s signalling. A, Western blotting analysis of the changes in ER stress signalling pathways in mBM‐MSCs treated with indicated concentrations of bortezomib for 24 h. Upper panel: One representative blot is shown of three independent experiments; lower panel: densitometric analysis of the Western blotting results. B, Western blotting analysis of the expression of Xbp1s, p21^Cip1^ and p27^Kip1^ in mBM‐MSCs treated with bortezomib in the presence of IRE1α inhibitor MKC3946 for 24 h. Upper panel: One representative blot is shown of three independent experiments; lower panel: densitometric analysis of the Western blotting results. C, Western blotting analysis of the expression of Atf4, p21^Cip1^ and p27^Kip1^ in mBM‐MSCs treated with bortezomib in the presence of PERK inhibitor GSK2606414 for 24 h. Upper panel: One representative blot is shown of three independent experiments; lower panel: densitometric analysis of the Western blotting results. D, Real‐time PCR analysis of the mRNA expression level of *p21^Cip1^* and *p27^Kip1^* in mBM‐MSCs treated with bortezomib (2.5 nM) in the presence of IRE1α inhibitor MKC3946 (10 nM) for 24 h. The values represent the mean ± SEM of three experiments, * *P* < .05, ** *P* < .01

To further investigate whether ER stress is involved in bortezomib‐induced G_0_/G_1_ phase arrest, we analysed the expression of key ER stress signalling‐related proteins, including the ER stress markers Grp78 and Chop, as well as three major regulators Xbp1s, Atf4 and Atf6, in response to bortezomib treatment. As shown in Figure 4A, bortezomib treatment significantly activated ER stress signalling, demonstrated by the increased expression levels of the ER stress markers Grp78 and Chop. More importantly, we observed that two ER stress signalling regulators Xbp1s and Atf4 were activated, demonstrated by higher‐level expression of Xbp1s and Atf4 by bortezomib.

To validate the regulatory relationship between the activation of ER stress signalling and the induction of p21^Cip1^ and p27^Kip1^, we used MKC3946 (an inhibitor of inositol‐requiring enzyme 1α (IRE1α)) and GSK2606414 (an inhibitor of double‐stranded RNA‐activated protein kinase [PKR]‐like ER kinase (PERK)) to block the bortezomib‐activated ER stress signalling pathways accordingly. As shown in Figure 4B, when the bortezomib‐induced Xbp1s was aborted by MKC3946, we also found a decrease in the expression of p21^Cip1^ and p27^Kip1^. However, when using GSK2606414 to block PERK‐Atf4 signalling, although we observed a marked decrease in Atf4, it had no significant effects on the expression of p21^Cip1^ and p27^Kip1^ (Figure 4C). More importantly, we further confirmed that the MKC3946‐aborted up‐regulation of *p21^Cip1^* and *p27^Kip1^* happened at the mRNA level, validated by real‐time PCR (Figure 4D). Given the potential effects of MKC3946 on the cells, we further analysed the changes of cell cycle and found that the combination of MKC3946 with bortezomib significantly decreased the percentage of S phase, but MKC3946 alone had no effects on the cell cycle distribution (Figure S2). These results strongly suggest that the activation of Xbp1s may be tightly associated with the expression of p21^Cip1^ and p27^Kip1^.

### 3.7 Enforced expression of XBP1s up‐regulates p21^Cip1^ and p27^Kip1^ and induces G_0_/G_1_ cell cycle arrest in mBM‐MSCs

**FIGURE 5:**
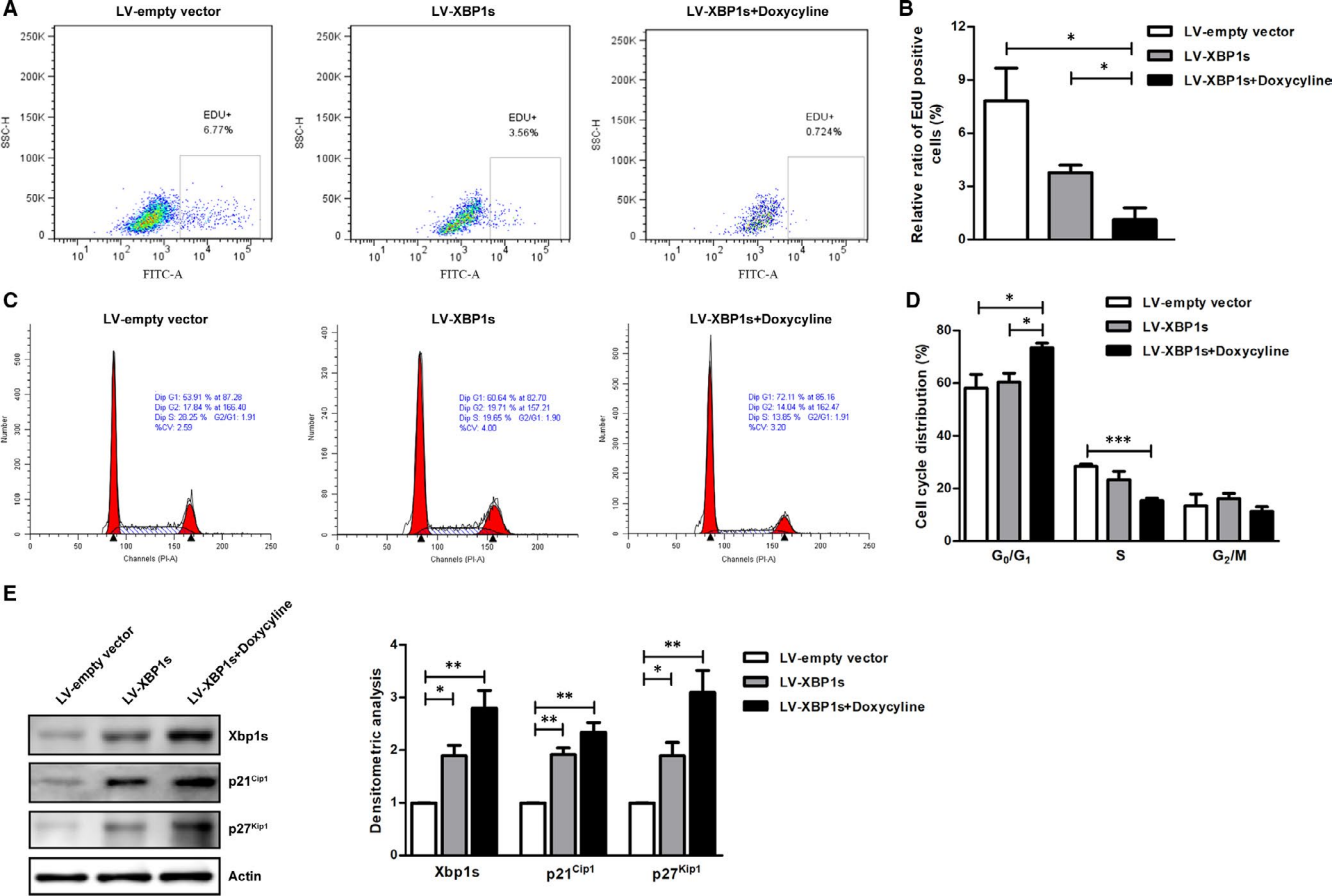
Overexpression of XBP1s in mBM‐MSCs inhibits cell proliferation and induces G_0_/G_1_ cell cycle arrest. A, One representative results of the flow cytometric analysis of the EdU incorporation in S phase of mBM‐MSCs infected with an inducible lentiviral system expressing XBP1s. B, Statistical analysis of the results of EdU incorporation assay. C, One representative results of the flow cytometric analysis of the effects of the enforced expression of Xbp1s on cell cycle. D, Statistic analysis of the cell cycle distribution in mBM‐MSCs with/without Xbp1s overexpression. E, Western blotting analysis of the effects of the enforced XBP1s expression on p21^Cip1^ as well as p27^Kip1^. Left panel: One representative blot is shown of three independent experiments; right panel: densitometric analysis of the Western blotting results. *P < 0.05, ** *P* < 0.01, n = 3

To further investigate the role of Xbp1s in cell cycle arrest, we used a Tet‐On lentiviral system to overexpress human spliced XBP1 in mBM‐MSCs. We found that enforced expression of XBP1s inhibited cell proliferation and induced G_0_/G1 cell cycle arrest in mBM‐MSCs (Figure 5A‐D). More importantly, it confirmed that the induction of p21^Cip1^ and p27^Kip1^ is accompanied by high‐level expression of XBP1s (Figure 5E).

### 3.8 Transcriptional regulation of p21^Cip1^ and p27^Kip1^ by Xbp1s

**FIGURE 6:**
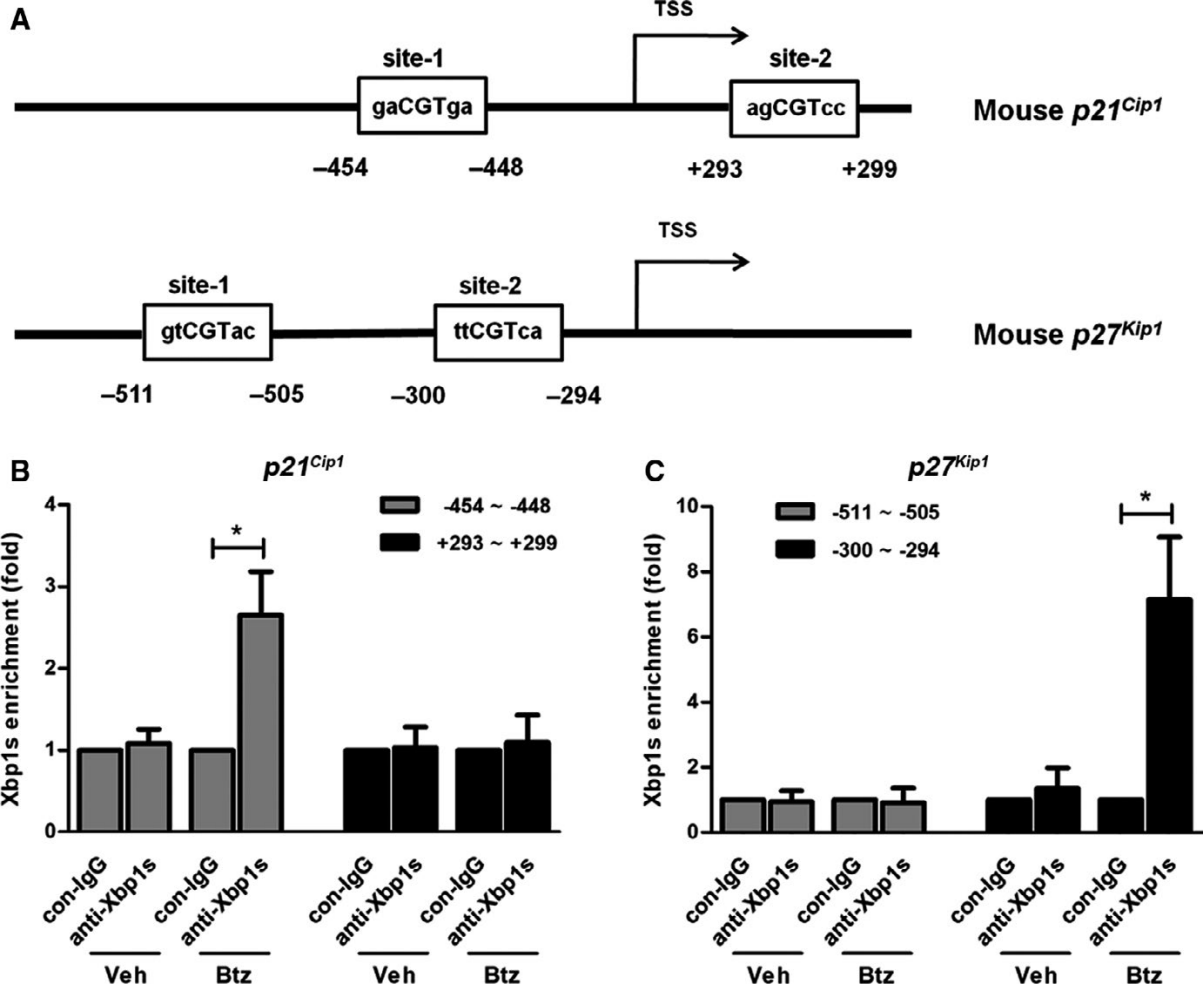
Xbp1s binds to the promoter of *p21^Cip1^* and *p27^Kip1^*. (A) Graphic representation of the putative Xbp1s binding sites in *p21^Cip1^* and *p27^Kip1^* promoter. Two putative Xbp1s binding sites were identified in the promoter of the *p21^Cip1^* and *p27^Kip1^* by searching for the Eukaryotic Promoter Database. (B‐C) Chromatin immunoprecipitation followed by real‐time PCR assay of Xbp1s binding in the *p21^Cip1^* and *p27^Kip1^* promoters in response to 0 and 2.5 nM bortezomib treatment for 16 h. Results are expressed as percentage of input. *P < 0.05 compared with control (n = 3)

To elucidate the potential transcriptional regulation of Xbp1s, we sought to determine whether Xbp1s binds to the *p21^Cip1^* and *p27^Kip1^* promoter. Prediction of transcription factor‐binding consensus sequences was carried out using the Eukaryotic Promoter Database (https://epd.epfl.ch/human/human_database.php?db=human) and identified two putative consensus binding sites for Xbp1s in both *p21^Cip1^* and *p27^Kip1^* promoter sequences from −2000 to +500 (Figure 6A). Consistently, ChIP analysis further confirmed the binding of Xbp1s to the ‘‐454 to −448’ site of the *p21^Cip1^* promoter and to the ‘−300 to −294’ site of the *p27^Kip1^* promoter (Figure 6B,C).

## 4 DISCUSSION

**FIGURE 7:**
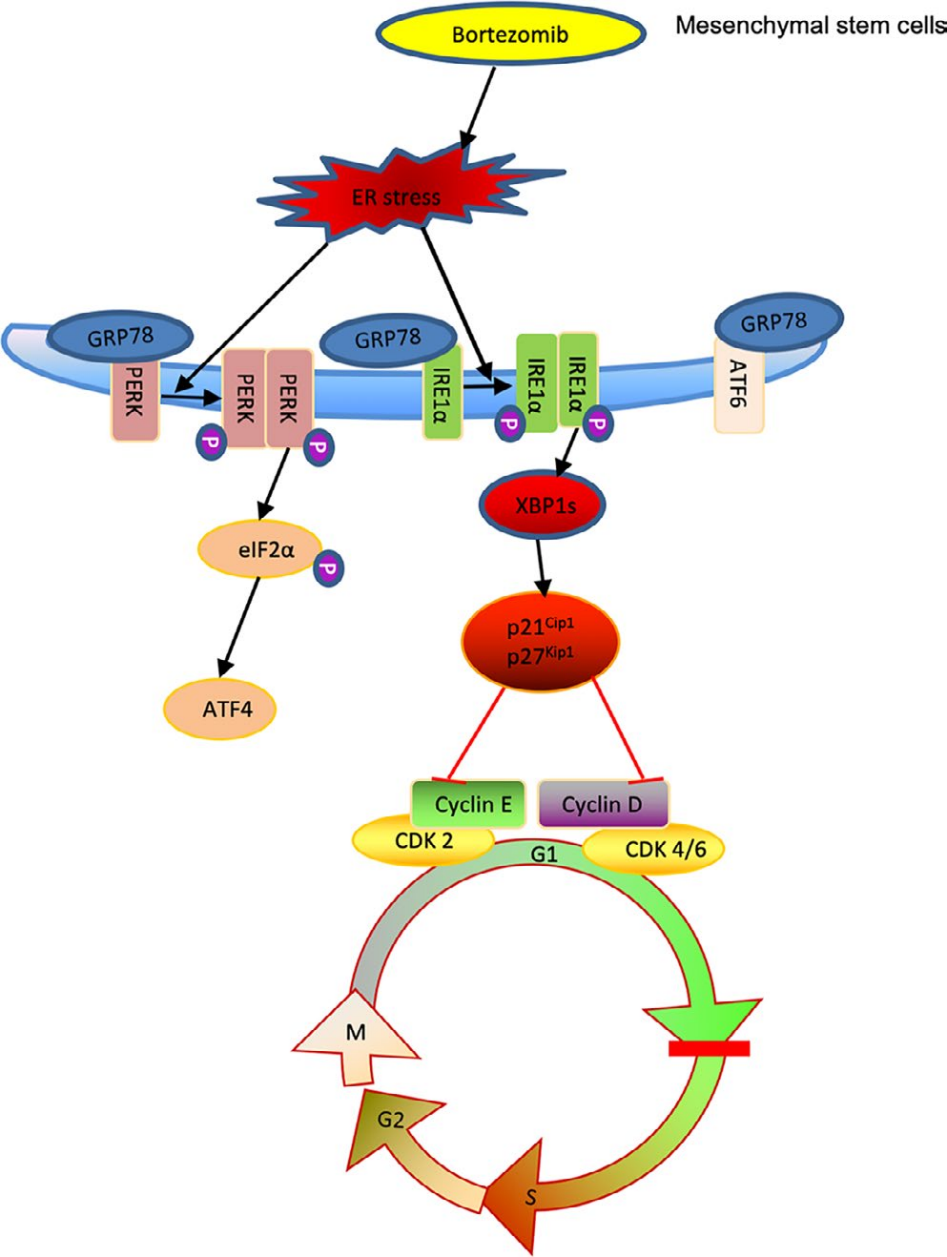
Diagrammatic presentation of the potential mechanism of cell cycle exit during bortezomib‐induced osteogenic differentiation of mBM‐MSCs

The development of multicellular organisms relies on the temporal and spatial control of cell proliferation and differentiation.^19^, ^22^, ^23^, ^24^ Developmental signals not only direct cell cycle progression but also set the frame for cell cycle regulation by determining cell type–specific cell cycle modes.^25^, ^26^ Usually, inhibition of the cell cycle is a requisite for terminal differentiation.^23^, ^25^, ^27^, ^28^ However, the precise cell cycle mechanisms for growth/differentiation transition remain unclear. In this study, we found that there exists a cell cycle exit that is mediated by the accumulation of CKIs p21^Cip1^ and p27^Kip1^ during bortezomib‐induced osteogenic differentiation of MSCs and further demonstrated that the induction of p21^Cip1^ and p27^Kip1^ was transcriptionally up‐regulated by the activated ER stress signalling regulator Xbp1s (Figure 7).

Bortezomib is a proteasome inhibitor of the 26S proteasome that plays a central role in protein degradation. The introduction of bortezomib has been a major breakthrough in the treatment of MM.^29^ Besides the anti‐MM activity, both preclinical and clinical data also substantiate that bortezomib plays a significantly beneficial role in bone formation.^30^ The increased osteoblast differentiation in BM hypothesizes one possible mechanism behind bone protection.^17^, ^31^, ^32^, ^33^, ^34^, ^35^ In the current study, by using mBM‐MSCs as an in vitro model, we demonstrated that bortezomib can induce osteogenic differentiation, validated by the markedly enhanced ARS staining. Our findings in mBM‐MSCs confirmed the previous report in human MSCs.^13^, ^36^ Meanwhile, EdU incorporation assay demonstrated that cell proliferation was almost entirely blocked by bortezomib. Cell cycle analysis further indicated that a G_0_/G_1_ phase arrest was induced by bortezomib in mBM‐MSCs. These findings strongly indicate a link between G_0_/G_1_ phase arrest and bortezomib‐induced differentiation.

Cell cycle progression is tightly governed by CDKs, which are the major regulators of the cell division cycle, activated by cyclin binding and inhibited by CKIs.^36^, ^37^ The close cooperation between this trio is necessary for ensuring orderly progression through or exit from the cell cycle. For this reason, we further studied the changes of cyclins, CDKs and CKIs in response to bortezomib treatment and found that the expression of G_0_/G_1_ phase‐related CDKs such as Cdk2 and Cdk4 was decreased by bortezomib. More importantly, the expression of p21^Cip1^ and p27^Kip1^ was observed to be increased significantly by bortezomib. Considering that p21^Cip1^ and p27^Kip1^ were extensively characterized as negative regulators of progression through G_1_ to S phase in mammalian cells, and several lines of evidence have suggested that p21^Cip1^ and p27^Kip1^ exert similar effects on cell cycle progression by mediating the inhibition of Cdk2 and/or Cdk4 activities,^38^, ^39^ it is reasonable to speculate that the induction of *p21^Cip1^* and *p27^Kip1^* may play an important role in the cell cycle exit induced by bortezomib.

It is known that p21^Cip1^ and p27^Kip1^ can inhibit cell cycle progression in response to numerous stimuli, but little is known about their involvement in proteasome inhibitor‐induced cell cycle changes. Recent studies of proteasome inhibition in MM cells revealed that the accumulation of unfolded proteins in the endoplasmic reticulum, referred to as ER stress, is considered the main mechanism of bortezomib‐induced apoptosis.^40^, ^41^, ^42^, ^43^ Normally, perturbation of endoplasmic reticulum homeostasis results in ER stress by the activation of a finely regulated programme defined as unfolded protein response (UPR). The mammalian ER stress consists of three canonical signalling pathways that are regulated by IRE1α‐XBP1, PERK‐Atf4 and Atf6.^44^ Focusing on the mechanisms of inducing p21^Cip1^ and p27^Kip1^, we further investigated whether the up‐regulation of p21^Cip1^ and p27^Kip1^ is related to the ER stress signalling activated by bortezomib. Firstly, we found that the up‐regulation of *p21^Cip1^* and *p27^Kip1^* occurred at the mRNA level. Next, we confirmed that bortezomib can activate both PERK‐Atf4 and IRE1α‐Xbp1s signalling pathways in mBM‐MSCs. Thirdly, we confirmed that Xbp1s other than Atf4 plays a major role in regulating *p21^Cip1^* and *p27^Kip1^* expression. More importantly, by performing ChIP assay, we demonstrated the direct interaction between Xbp1s and the promoter of *p21^Cip1^* and *p27^Kip1^*, further supporting the role of Xbp1s in transactivating the transcriptional activity of the *p21^Cip1^* and *p27^Kip1^*.

Xbp1 is a bZIP (basic‐region leucine zipper) transcription factor that interacts specifically with the conserved X2 boxes of major histocompatibility complex class II gene promoters.^45^ *Xbp1* can yield two isoforms: unspliced Xbp1 (Xbp1u) and spliced Xbp1 (Xbp1s). In response to ER stress, the mRNA of Xbp1u is spliced to generate Xbp1s, which is considered as the active form, playing a pivotal role in ER stress signalling. Nonetheless, Xbp1u has also been shown to inhibit Xbp1s‐mediated effects. For example, Xbp1u has been demonstrated to down‐regulate the expression of *p21^Cip1^* by negatively inhibiting the p53/p21 axis.^46^ Moreover, it has been indicated that Xbp1s is essential for bone morphogenic protein 2‐induced osteoblast differentiation through up‐regulating the transcription of Osterix, which is an osteoblast‐specific transcription factor.^47^ We further demonstrated that Xbp1s plays central roles in regulating several osteogenic differentiation‐related genes in response to bortezomib stimuli (data not shown). Focusing on the effect of Xbp1s in the cell cycle, we further showed that forced expression of XBP1s in mBM‐MSCs can directly trigger the accumulation of p21^Cip1^ and p27^Kip1^. Meanwhile, we observed that forced expression of Xbp1s can drive mBM‐MSC differentiation into osteoblasts (data not shown).

In addition to the well‐known function of CKIs in cell cycle control, it is becoming increasingly apparent that CKIs also play indispensable roles in processes such as transcription and epigenetic regulation. Both p21^Cip1^ and p27^Kip1^ are known to interact with a range of transcription factors involved in modulating the expression of numerous genes in various biological processes.^48^ In this regard, one limitation of this study is that we cannot conclude whether the up‐regulated *p21^Cip1^* and *p27^Kip1^* directly stimulate the expression of osteogenic‐related genes. Secondly, we cannot conclude whether p21^Cip1^ and p27^Kip1^ play redundant roles in this process. For example, although both p21^Cip1^ and p27^Kip1^ proteins were induced during erythroid differentiation, only p27^Kip1^ is associated with the inactivation of Cdk2, and p21^Cip1^ may have a function independent of growth arrest during erythroid differentiation.^49^ In myeloid leukaemia cells, p21^Cip1^ and p27^Kip1^ have been demonstrated to induce distinct cell cycle effects and differentiation programmes.^39^

## 5 CONCLUSIONS

In this study, we demonstrated that bortezomib‐induced p21^Cip1^ and p27^Kip1^ are required for cell cycle exit during osteogenic differentiation and that induction of *p21^Cip1^* and *p27^Kip1^* by bortezomib is transcriptionally regulated by activation of the ER stress signalling pathway Ire1α/Xbp1s. These findings may provide valuable information enabling a better understanding of the mechanisms underlying proteasome inhibitor‐induced osteogenic differentiation of MSCs.

## CONFLICT OF INTEREST

The authors report no conflict of interest.

## AUTHOR CONTRIBUTIONS

JH and DZ designed the experiments, analysed and interpreted the experimental results and wrote the manuscript. RF, LL, LL, YM and NL performed most of the experiments and analysed the experimental data. PC and RAW carried out Western blotting and real‐time PCR analysis. BW made substantial contributions to the conception and design of the study and revised the manuscript. All authors read and approved the manuscript.

## Supporting information

Fig S1

Fig S2

Table S1

Table S2

## Data Availability

The data used to support the findings of this study are available from the corresponding author upon request.
