# Supplementary material for: Cell cycle exit during bortezomib‐induced osteogenic differentiation of mesenchymal stem cells was mediated by Xbp1s‐upregulated p21Cip1 and p27Kip1
Source: J Cell Mol Med. 2020 Jul 6;24(16):9428–38. doi: 10.1111/jcmm.15605 (PMC7417721; doi:10.1111/jcmm.15605)
Supplement: Supplementary file 1 — Fig S1 [file JCMM-24-9428-s001.docx]

**Supplementary Figure 1:**


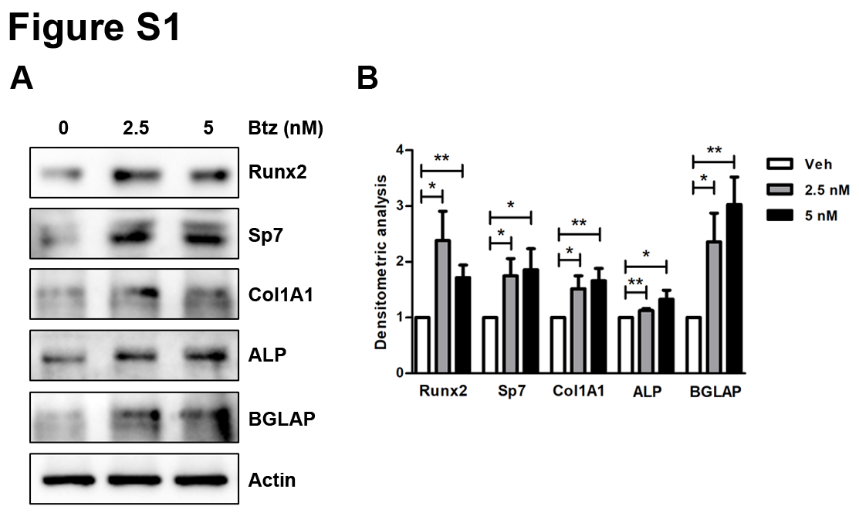


**Figure S1.** Bortezomib induces the expression of osteogenesis-related proteins. (A) Western blotting analysis of the changes of the osteogenesis-related proteins in mBM-MSCs treated with indicated concentrations of bortezomib for 24 h. (B) Densitometric analysis of the western blotting results of three independent experiments (* *P* < 0.05; ** *P* < 0.01).
