# Supplementary material for: Cell cycle exit during bortezomib‐induced osteogenic differentiation of mesenchymal stem cells was mediated by Xbp1s‐upregulated p21Cip1 and p27Kip1
Source: J Cell Mol Med. 2020 Jul 6;24(16):9428–38. doi: 10.1111/jcmm.15605 (PMC7417721; doi:10.1111/jcmm.15605)
Supplement: Supplementary file 2 — Fig S2 [file JCMM-24-9428-s002.docx]

**Supplementary Figure 2:**


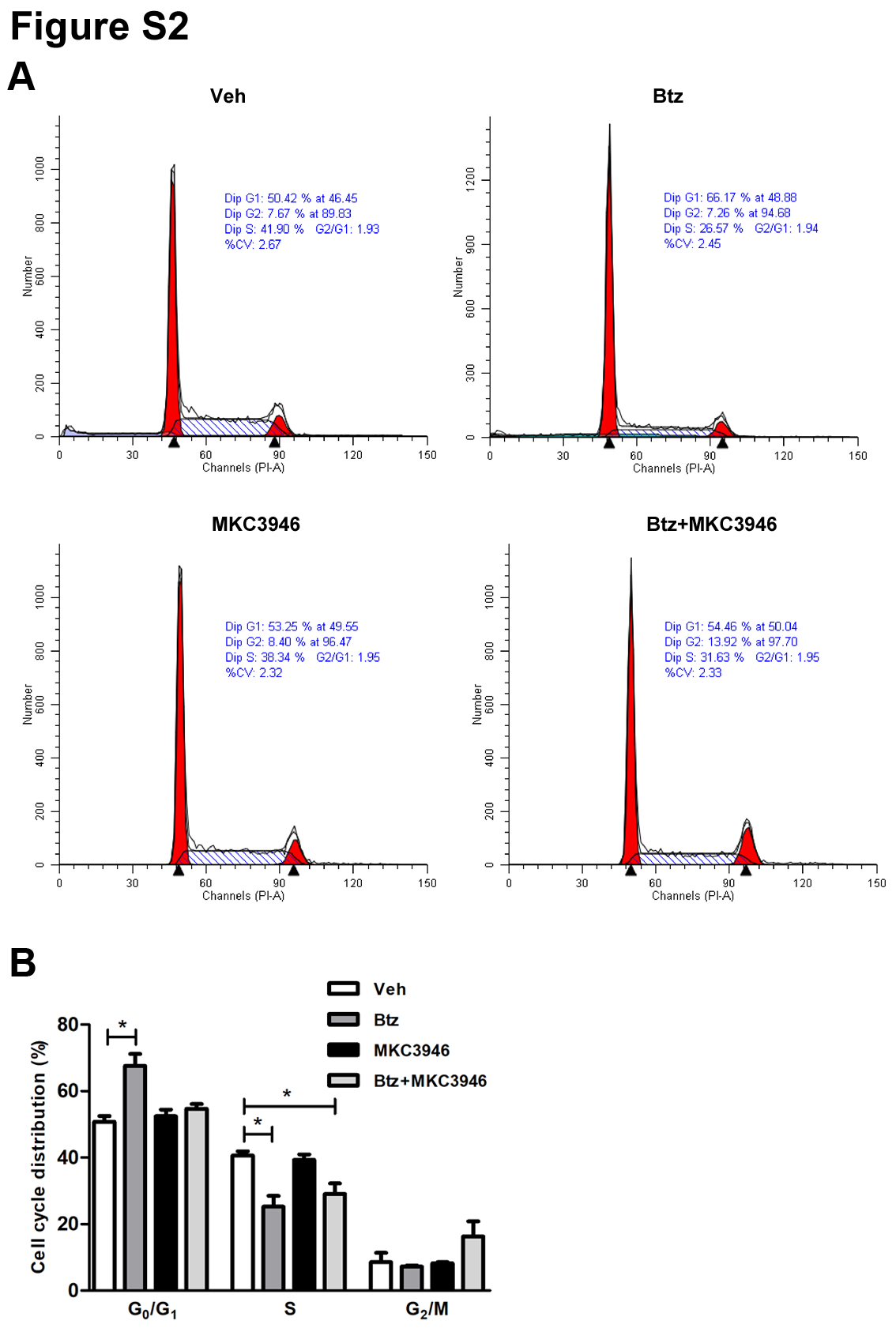


**Figure S2.** The effects of bortezomib and MKC3946 on cell cycle. (A) mBM-MSCs were treated with vehicle, 2.5 nM bortezomib, 10 nM MKC3946 and the combination for 24 h, then the cells were collected for cell cycle analysis using flow cytometry. (B) Statistical analysis of cell cycle distribution. Data presented as mean  ±  SEM of three independent experiments (* *P* < 0.05).
