# Supplementary material for: Cell cycle exit during bortezomib‐induced osteogenic differentiation of mesenchymal stem cells was mediated by Xbp1s‐upregulated p21Cip1 and p27Kip1
Source: J Cell Mol Med. 2020 Jul 6;24(16):9428–38. doi: 10.1111/jcmm.15605 (PMC7417721; doi:10.1111/jcmm.15605)
Supplement: Supplementary file 3 — Table S1 [file JCMM-24-9428-s003.docx]

**Supplementary Table 1. Primer sequences used for Realtime PCR analysis**

| **Gene symbol** | **Gen Bank Accession no.** | **Primer set sequence (5’->3’)** | **Amplicon size (bp)** |
| --- | --- | --- | --- |
| *Beta-actin* (m) | NM_007393.5 | Forward:  GCGACAGCAGTTGGTTGGAG  Reverse:  TTTGGGAGGGTGAGGGACTTC | 165 |
| *p21^Cip 1^* (m) | NM_007669.5 | Forward:  GCGACAGCAGTTGGTTGGAG  Reverse:  TTTGGGAGGGTGAGGGACTTC | 373 |
| *p27^Kip1^* (m) | NM_009875.4 | Forward:  GCGACAGCAGTTGGTTGGAG  Reverse:  TTTGGGAGGGTGAGGGACTTC | 284 |
