# Supplementary material for: Cell cycle exit during bortezomib‐induced osteogenic differentiation of mesenchymal stem cells was mediated by Xbp1s‐upregulated p21Cip1 and p27Kip1
Source: J Cell Mol Med. 2020 Jul 6;24(16):9428–38. doi: 10.1111/jcmm.15605 (PMC7417721; doi:10.1111/jcmm.15605)
Supplement: Supplementary file 4 — Table S2 [file JCMM-24-9428-s004.docx]

**Table 1. Primer sequences used for ChIP assay**

| **Gene symbol** | **Primer set** | **Location** | **Primer set sequence (5’->3’)** | **Amplicon size (bp)** |
| --- | --- | --- | --- | --- |
| *p21^cip1^* (m) | Site 1 | -540/-377 | Forward:  TGGTCTCCATCGGAATAGGT  Reverse:  TGTTTGCCTAACTTGCTGGA | 164 |
|  | Site 2 | +187/+417 | Forward:  TGTGTGTGGTGATGAGTGGA  Reverse:  TCCAAGGACTGGAAGAGTGG | 231 |
| *p27^Kip1^* (m) | Site 1 | -580/-481 | Forward:  GGCCGTTTGGCTAGTTTGTT  Reverse:  CTGGTCGCGTGACTACTCG | 100 |
|  | Site 2 | -357/-203 | Forward:  AGACCAATGGAGCTCCTCCT  Reverse:  GAGTCGGGACAAGGGATGA | 155 |
